# Investigation on the Influence of Process Parameters on the Mechanical Properties of Extruded Bio-Based and Biodegradable Continuous Fiber-Reinforced Thermoplastic Sheets

**DOI:** 10.3390/polym15183830

**Published:** 2023-09-20

**Authors:** Maximilian Lang, Benedikt Neitzel, Shiva MohammadKarimi, Florian Puch

**Affiliations:** 1 Plastics Technology Group, Faculty of Mechanical Engineering and Thuringian Center of Innovation in Mobility, Technische Universität Ilmenau, 98693 Ilmenau, Germany; benedikt.neitzel@tu-ilmenau.de (B.N.); shiva.mohammadkarimi@tu-ilmenau.de (S.M.); florian.puch@tu-ilmenau.de (F.P.); 2 Thüringisches Institut für Textil- und Kunststoff-Forschung Rudolstadt e.V., 07407 Rudolstadt, Germany

**Keywords:** continuous fiber-reinforced thermoplastic sheets, polymer composite, biopolymer, natural fiber, fiber-reinforced polymer, direct extrusion

## Abstract

The use of bio-based and biodegradable matrix materials in fiber-reinforced polymers (FRPs) is an approach to reduce the consumption of fossil resources and the amount of polymer waste. This study aims to assess the influence of the process parameters on the resulting mechanical properties of extruded bio-based and biodegradable continuous fiber-reinforced thermoplastics (CFRTPs) in the form of sheets. Therefore, the impregnation temperature during the production of PLA/flax fiber composites is varied between 220 °C and 280 °C, and the consolidation pressure, between 50 bar and 90 bar. A design of experiments approach is used. Fiber contents of 28.8% to 34.8% and void contents of 6.8% to 15.5% are determined for the composites by optical measurements. To assess the mechanical properties, tensile tests are performed. Using the evaluation software Minitab, a strong negative influence of the consolidation pressure on the tensile modulus and the tensile strength is observed. Increasing the pressure from 50 bar to 90 bar results in a reduction in the tensile modulus of 50.7% and a reduction in the tensile strength of 54.8%, respectively. It is assumed that this is due to fibers being damaged by the external force exerted onto the materials during the consolidation process in the calender. The influence of the impregnation temperature on the mechanical properties cannot be verified.

## 1. Introduction

### 1.1. Motivation

Continuous fiber-reinforced thermoplastics (CFRTPs) are a key material for lightweight applications. This material group combines low specific gravities with excellent mechanical properties. Therefore, composites with high strength-to-weight and stiffness-to-weight ratios can be created. This in combination with other beneficial properties like resistance against fatigue and corrosion shows why CFRTPs are established in various industrial sectors [1]. Especially in the transportation sector, there is a high potential for the use of lightweight components, e.g., Japan’s Ministry of Transport reported that a weight reduction in a single automobile of 100 kg reduces its CO_2_ emissions by 20 g/km on average [2].

Concerns when using synthetic polymers are, among others, the consumption of fossil resources and the handling of polymer waste. The amount of chemicals produced by the petrochemical industry used in polymer materials is about 80% by weight [3]. According to Geyer, Jambeck and Law the amount of polymers, synthetic fibers and additives produced between 1950 and 2015 was 8.3 Gt. In 2015, 6.3 Gt of those products were already declared as waste, of which 9% have been recycled and 12% were incinerated. The remaining 79% were found in landfills or the natural environment. It is projected that the amount of polymer waste will rise up to 12 Gt by 2050 [4]. The most concerning part is the waste that has entered the environment. The model Eriksen et al. created shows that there are approximately 5.25 trillion polymer particles in the world’s oceans. They weigh about 268,940 t and are not only an ecological burden but can also enter the human food chain through the consumption of fish and other seafood, where correlating health issues cannot be ruled out at this point [5,6].

The use of biodegradable polymers facilitates a reduction in the annually rising amount of polymer waste. Furthermore, life-cycle assessments show that bio-based polymers can be less energy consuming in production than their synthetic counterparts, while emitting less greenhouse gas and reducing the consumption of fossil resources. Sustainability can be increased even further by incorporating bio-based additives. The comparison of natural fibers, e.g., flax fibers, and synthetic fibers, e.g., glass fibers, shows a lower environmental impact and lower cost for post-life handling on the natural fiber side [7,8,9]. In addition, the lower density of flax fibers allows for a further weight reduction in the structural elements in which glass fibers are usually used, e.g., panels in automobiles [10]. In order to incorporate natural materials into technical products, they have to be processible in an efficient and cost-effective manner. Continuous production processes for CFRTP sheets like the direct extrusion process meet those criteria. To produce high quality composites, the process parameters as well as their influence on the morphology and mechanical properties are investigated.

### 1.2. Continuous Fiber-Reinforced Thermoplastics (CFRTPs)

CFRTP sheets are flat and consist of fully impregnated fibers and a thermoplastic matrix. The greatest advantage of these composites is the ability to be reheated and formed to create components with complex shapes [11,12]. The production process of CFRTP sheets includes the impregnation of the fiber with matrix material, the consolidation of the materials under pressure and the solidification of the matrix [12,13]. The temperature and the process pressure as well as the mass flow of polymer material have a strong influence on the process and the quality of the composite. The amount of polymer material that is applied on the fiber material during extrusion depends on the haul-off speed and the throughput of polymer. Given that the throughput is constant, the haul-off speed becomes a process relevant parameter [12,13].

High processing temperatures reduce the melt viscosity η of the polymer and improve the impregnation process [14]. The aim of the consolidation process is to combine the fabric layers with the thermoplastic matrix material to create a composite with a low void content. Therefore, an external pressure is applied. The amount of pressure and the given consolidation time are key parameters for this process [13,15,16]. The consolidation time must be long enough to allow the molten polymer to advance into the bundles before it starts to solidify [13,17].

### 1.3. Impregnation by Thermoplastic Polymers

Direct extrusion describes the continuous process of directing a long-fiber component through a heated die and impregnating the fibers with a polymer melt to produce CFRTP sheets. This results in a reduction in process- and downtime as well as an increase in energy efficiency through an isothermal operation of the die [18].

The impregnation of the reinforcement fibers with the polymer matrix material is the most important step to produce CFRTPs. This complex process is influenced by various parameters. After the materials are brought into contact with one another, the process pressure is applied to combine them. During this step, the fabric is compressed. The polymer starts to slowly penetrate the fabric. At this point, the air inside the fabric is forced out, and the fabric starts to relax. The full relaxation takes longer than the impregnation with matrix material [13].

The impregnation of the volume between fiber bundles is considered as macro-impregnation, while micro-impregnation is the impregnation of the volume inside the fiber bundles, which takes place with a delay. The applied polymer affects the geometry of the fiber bundles in terms of higher undulation angles of the bundles and the compression of the bundles. The individual fibers are compressed to the densest packaging. The microscopic deformations cause a dissipation of fluid energy, which is not available for the impregnation process. After the macro-impregnation is completed, the polymer-flow-induced pressure on the fiber bundles is much lower than during the macro-impregnation process. This is when the fiber relaxation starts. The fibers expand, which allows the polymer to penetrate the bundles. This expansion is supported by the polymer flow through the capillaries between the fibers. Simultaneously, the permeability inside of the bundles increases [13].

The Kozeny–Carman equation is based on Darcy’s law and allows to calculate the bundle permeability K_zz_ (1). The permeability perpendicular to the fiber direction is influenced by the fiber radius r_f_ and the fiber volume content V_f_. The Kozeny constant is described by k_zz_ [17].

(1) $$K_{zz}=\frac{r_{f}^{2}}{4k_{zz}}\frac{{\left(1-V_{f}\right)}^{3}}{V_{f}^{2}}$$

The maximum fiber content depends on the fiber packing. For a quadratic fiber packing, the maximum fiber volume content is 78.5%, and for a hexagonal fiber packing, it is 90.7%. In reality, mixed configurations of quadratic and hexagonal fiber packing occur [14,17].

The Kozeny–Carman equation results in a uniform permeability, as it is present in a porous medium. In a fiber bundle, the permeability longitudinal to the fibers is higher than in the perpendicular direction. For the described impregnation process, the permeability perpendicular to the fibers $K_{zz}^{\prime }$ is relevant and can be calculated using the Kozeny constant modified by Gutowski $k_{zz}^{\prime }$ (2) [17].

(2) $$K_{zz}^{\prime }=\frac{r_{f}^{2}}{4k_{zz}^{\prime }}\frac{{\left(\sqrt{\frac{V_{fmax}}{V_{f}}-1}\right)}^{3}}{\left(\frac{V_{fmax}}{V_{f}}+1\right)}$$

Another parameter that influences the impregnation is the polymer viscosity. Following Equation (3), the viscosity can be described as the resistance of a fluid against a shear stress. The viscosity is temperature- and shear-rate-dependent and therefore variable. The shear rate for calendered thermoplastic polymers is usually lower than 100 1/s [14,19]. The polymer viscosity can be calculated according to Ostwald and de Waele using the flow index n as follows (4) [19]:

(3) $$\eta =\frac{\tau }{\dot{\gamma }}$$

(4) $$\eta =K\;{\times \;\dot{\gamma }}^{n-1}$$

### 1.4. Influences of Voids and Undulation

The inclusion of voids affects various properties of the CFRTP [20,21]. Lower resistance against interlaminar shear and longitudinal compressive and transversal tensile stress are the result of a high void content [22]. The voids provide a critical point for stresses. Apart from the void content, the void properties regarding form, diameter and distribution are relevant [23]. The main reason for void formation in thermoplastic composites is trapped air during the impregnation process. The high melt viscosity of thermoplastic materials hinders the air removal and the ability to impregnate the inside of the fiber bundle [23]. According to research, the process parameters temperature, pressure and impregnation time are varied to reduce the number of voids inside of the fiber bundle (micro-voids) and the ones in between the bundles (macro-voids) [22,23]. Voids can also be the result of gases that build up during the production process or water residing in the materials, which evaporates at the high temperatures present during processing [23].

Fiber undulation is a characteristic of most woven fiber fabrics. The arrangement and overlap of the bundles result in curved fibers. Hence, fibers are no longer ideally oriented, so multidirectional stresses arise from unidirectional strains. Therefore, the mechanical properties are noticeably reduced [22,24]. The local overlaps are labeled weaving points or knots.

The fiber distribution in an ideal composite is assumed to be homogeneous. A composite with an inhomogeneous distribution shows areas with significantly lower fiber content. These areas are dominated by matrix material and can weaken the composite [22]. In terms of tensile strength, the matrix is much weaker than the reinforcement material. The matrix dominated areas are the result of fiber displacement due to the process pressure and the polymer melt. The displacement can be reduced by increasing the temperature to reduce the melt viscosity of the matrix or by lowering the process pressure [22].

### 1.5. Research Approach

This study aims to assess the influence of process parameters on the mechanical properties of bio-based and biodegradable continuous fiber-reinforced thermoplastic sheets using a continuous extrusion-based manufacturing process. The continuous process poses additional challenges for the manufacturing of composites with a low void content compared to more commonly used discontinuous processes, e.g., film-stacking, due to shorter impregnation times [25,26]. According to research, great mechanical properties are based on a high fiber and a low void content for CFRTPs [22,23]. Fiber and void content can be optimized through the process parameters. To quantify the influence of the process parameters on the mechanical properties for a bio-based material combination, experiments are conducted where the process parameters are varied, and the resulting fiber contents, void contents and mechanical properties are determined. Therefore, a measuring method is chosen which allows to determine fiber and void content for the utilized materials. A design of experiments is implemented, and suitable ranges for process parameters are determined. After manufacturing, the samples are examined, and correlations between the results and the process parameters are assessed.

## 2. Materials and Methods

### 2.1. Materials

#### 2.1.1. Fibers

As the reinforcing component, a flax fiber fabric, ampliTex 5040 from Bcomp Ltd., Fribourg, Switzerland, was used. Flax fibers are obtained from the stem of the flax plant, from which bundles are obtained which contain multiple fibers held together by a pectin middle lamella. The flax plant belongs to the family of Linaceae, which is a common plant and therefore highly available [27,28]. The used fabric is a 2/2 twill weave, which allows a better utilization of the mechanical properties of the fiber due to less weave points and therefore less fiber undulation. The fabric has a mass per unit area of 300 g/m^2^ and a 300-tex yarn with no twist. The density of the fiber material is 1.47 g/cm^3^. Tests of the mechanical properties of the fiber showed a tensile modulus of 62 GPa and an elongation at break of 1.3% to 1.4%. Thermogravimetric analysis (TGA) revealed an onset decomposition temperature of 243.5 °C.

#### 2.1.2. Matrix

As matrix material polylactic acid (PLA), Ingeo Biopolymer 2003D produced by NatureWorks, was used. The mechanical properties of the PLA showed a tensile modulus of 3.5 GPa. At break, the material has a tensile strength of 53 MPa and an elongation of 6%. The thermal properties of the PLA are provided in Table 1. In addition, a TGA was conducted to determine the decomposition temperature, revealing an onset decomposition temperature of 349 °C.

### 2.2. Methods

#### 2.2.1. Description of the Used Direct Extrusion Process

**Figure 1:**
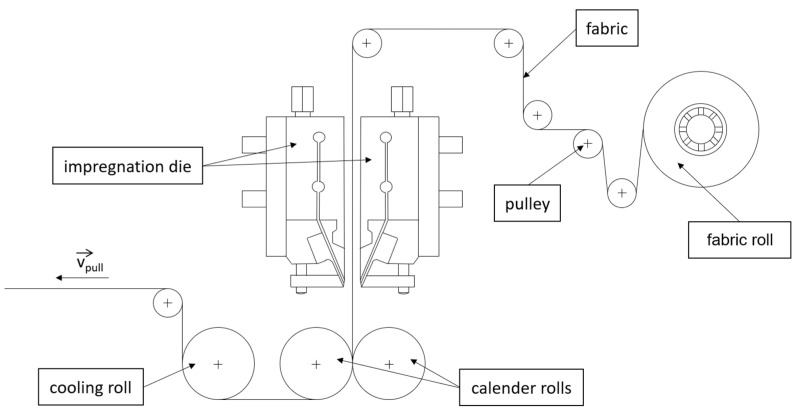
Schematic sketch of the used direct extrusion system.

Figure 1 depicts the system used to produce the CFRTP. In total, it consists of three segments, namely the supply unit, the impregnation unit and the cooling unit.

The supply unit consists of a twin-screw extruder which provides the polymer material and a rotating bar on which the flax fabric is placed. The screws have a diameter D of 25 mm and a length of 1.125 mm (45 × D). The amount of polymer granule used is regulated via an integrated scale and a shaking plate. The polymer is fed into the extruder where it is melted and afterward transported to the impregnation die via melt pumps. The mass flow of polymer is controlled via the control terminal and is measured in kg/h. In order to work continuously, fiber fabric in the form of rolls of 50 m in length and 0.25 m in width is used. The rolls are fixated and afterward unrolled. Multiple pulleys are used to redirect and apply tension to the fabric before feeding it to the impregnation die.

The impregnation unit consists of two heated dies, between which the fibers are preimpregnated with the matrix material. The two identical dies are fed with polymer which is distributed over the width of the fabric with a constant volume flow rate. Both dies are heated separately to keep the polymer at the needed impregnation temperature and avoid solidified material inside of the die. The impregnation dies are oriented vertically to distribute the matrix material on both sides of the fabric evenly and to avoid polymer becoming detached from the bottom by gravitation.

The preimpregnated fabric is directly transferred to the calender rolls which apply the impregnation and consolidation pressure. The preimpregnated fabric is pulled through the rolls to create an impregnated and consolidated composite. Both rolls are heated to facilitate the separation of the roll and composite. During extrusion, polymer accumulates in front of the calender rolls. This accumulation is crucial for an even impregnation and can be controlled via the haul-off speed and the polymer throughput.

Following the consolidation rolls, the composite is transferred to the cooling unit. The composite is directed over a chill roll and afterward redirected via pulleys. The matrix material is cooled down and collected.

#### 2.2.2. Parameter Variation and Design of Experiments

To determine the influences of the process parameters on the composite properties, a design of experiments experimental plan is used. Preliminary experiments showed that suitable and stable impregnation temperatures range from 220 °C to 280 °C, and process pressures, from 50 bar to 90 bar. The investigated experimental points (EPs) are shown in Table 2.

**Figure 2:**
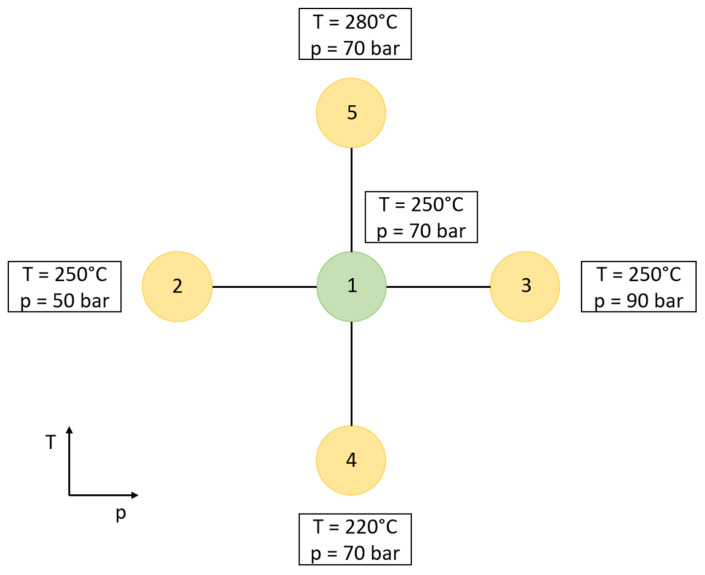
Experimental plan with a star formation.

The chosen experimental points are arranged in a star formation. EP 1 constitutes the center from where temperature and pressure are varied separately to determine the influence of single parameters. Figure 2 depicts the arrangement of the experimental points as well as the set values for temperature and pressure.

Aside from the processing temperature and the consolidation pressure, a polymer throughput of 8 kg/h and a haul-off speed of 0.8 m/min were chosen. This combination of parameters ensures that an optimal amount of polymer melt is applied onto the fabric, which is enough to fully impregnate the fabric without noticeably displacing the fibers or damaging them.

The haul-off speed is chosen regarding the required amount of polymer material, impregnation time and thermal exposure. Bourmaud et al. showed that too much thermal exposure reduces the mechanical properties of flax fibers significantly. They exposed a flax fiber fabric to 250 °C for eight minutes and determined a reduction in strength to 40% of the initial strength [29]. In our experiments, the temperatures reach up to 280 °C, which is why the exposure time should be short. Given the chosen haul-off speed, the fibers are exposed directly to the heat for 45 s, which is considered acceptable.

The heated calender rolls were set to a temperature of 70 °C and the following cooling roll to 50 °C. Higher temperatures are not possible because the PLA matrix does not solidify sufficiently and sticks to the calender rolls, destroying the CFRTP. The chosen settings allow the matrix to solidify in a controlled manner.

#### 2.2.3. Fiber and Void Content

The fiber and void content of the PLA/flax fiber composites cannot be determined via calcination as it is common for glass- or carbon-fiber-reinforced thermoplastics. The used PLA showed an onset temperature of 349 °C. The onset temperature of the flax fibers was 243.5 °C. Hence, the fibers cannot resist the calcination process. Consequently, an optical analysis was chosen.

After the CFRTPs were produced, ten samples of 40 mm in length and 40 mm in width were cut out. The samples were embedded in a thermoset cut, grinded, polished and examined with a microscope, Keyence VHX-7000, which allowed the analysis of the samples with a magnification of 500 times.

The micrographs were examined via an evaluation software which allowed to determine the surface areas of the composite A_comp_ and the weft thread A_weft_. The mean surface area of the weft thread was determined separately. Therefore, 20 bundles of 10 cm length were collected from the fabric and weighed. The volume of the fibers was calculated given the density of 1.47 g/cm^3^. With the volume and sample length determined, a mean surface area of 210,510.20 μm^2^ per bundle A_warp_ was calculated. Per sample, the area around four bundles was examined. The fiber content $\varphi_{F}$ was determined as follows (5) and (6):

(5) $$A_{F}=A_{weft}+4\times A_{warp}$$

(6) $$\varphi_{F}=\frac{A_{F}}{A_{comp}}$$

The void content X_p_ was determined based on the fiber content. Given the fiber content, a theoretical composite density $\rho_{th}$ was calculated. The measured density $\rho_{meas}$ differed from the theoretical value. This difference is due to voids inside the real composite. Based on both densities, the void content was determined as follows (7), (8):

(7) $$\rho_{th}=\varphi_{F}\rho_{F}+(1-\varphi_{F})\rho_{M}$$

(8) $$X_{P}=1-\frac{\rho_{meas}}{\rho_{th}}$$

The used method considers the matrix material, the warp thread and the weft thread as continuous objects regarding the depth of the samples. This assumption allows to determine fiber and void content by 2D images. However, the weft thread cannot be examined as precisely as the warp thread. Additionally, the fiber undulation cannot be recognized in one 2D image. To evaluate the deviations resulting from the assumption, CFRTP sheets consisting of a glass fiber plain weave and a PP matrix were produced and examined by calcination and the optical method, respectively. The sheets were produced at two experimental points. The experimental points as well as the resulting fiber and void contents are given in Table 3. The samples for calcination were weighed, and their density was measured using a scale for density determination, Sartorius Cubis MSU224S. After that, they were placed in a furnace, where they resided for 1.5 h at a temperature of 550 °C to fully decompose the polymer matrix. The remaining fibers were weighed again. Using the determined masses and densities, the fiber content, the theoretical composite density and the void content were calculated following DIN EN ISO 1172 [30].

The results showed only small deviations between the methods. Therefore, the described optical method was used for the analysis of the PLA/flax composites.

#### 2.2.4. Mechanical Properties

**Figure 3:**
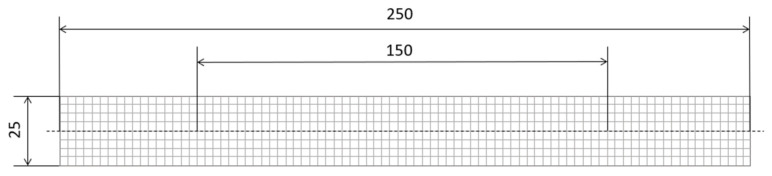
Sample for tensile tests with the dimensions and measurement length.

The mechanical properties of the CFRTP sheets were determined via tensile tests. Samples were taken from the sheets so that the tests could be conducted lengthwise to the warp thread. The samples had a rectangular shape of 25 × 250 mm according to DIN EN ISO 527–4 [31,32]. The tensile tests were conducted using a tensile testing machine, Shimazu AG-50 kNXplus, and a measurement length of 150 mm as well as a traverse speed of 2 mm/min. Figure 3 shows a sample with the dimensions and measurement length [31,32].

In order to measure the elongation ε of the sample, an elongation instrument was used, which was attached to the sample via springs, allowing for a direct measurement of the elongation of the composite sample.

To determine the mechanical properties, the thickness of the samples was measured and added into the calculation software. The software tracked the elongation and tensile tension and determined the tensile modulus, the tensile strength and the elongation at break. The tensile modulus E was determined according to Hooke’s law by examining the stress values σ in the area of 0.05% and 0.25% elongation, as shown in Equation (9).

(9) $$E=\frac{∆\sigma }{∆\varepsilon }=\frac{\sigma_{0.25\%}-\sigma_{0.05\%}}{\varepsilon_{0.25\%}-\varepsilon_{0.05\%}}$$

## 3. Results

### 3.1. Fiber and Void Content

Fiber and void content influence the mechanical properties significantly. Hence, these parameters were determined and compared to the tensile properties. The results of the fiber and void content analysis are shown in Table 4.

**Figure 4:**
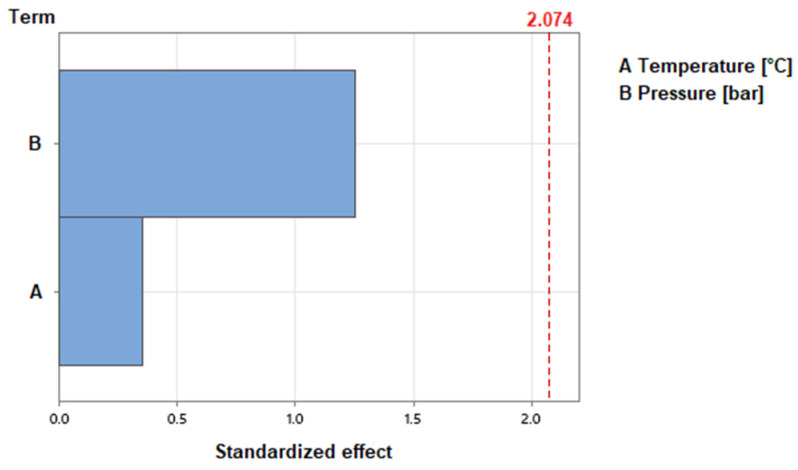
Standardized effects of the process parameters on fiber content, 95% confidence interval.

The results showed only small differences in fiber content for the different process parameters. For different temperatures, the fiber content changes with a maximum of 3.7% between the EPs 250 °C and 280 °C. The increase in the pressure results in a slight increase in the fiber content of 3.8% in total. Because the standard deviations of single experimental points are greater than the differences between the data points, the process parameters are examined individually for statistical relevance. The results are shown in Figure 4. The standardized effect of the process parameters on the fiber content is depicted as bars. The standardized effect of the parameter must reach the calculated threshold of 2.074 to consider its influence as statistically relevant. To calculate this threshold, a confidence interval of 95% is used. The figure shows that neither temperature nor pressure have an influence on the fiber content that reaches statistical relevance. Therefore, the fiber content is not significantly influenced by varying the process parameters for CFRTP production.

**Figure 5:**
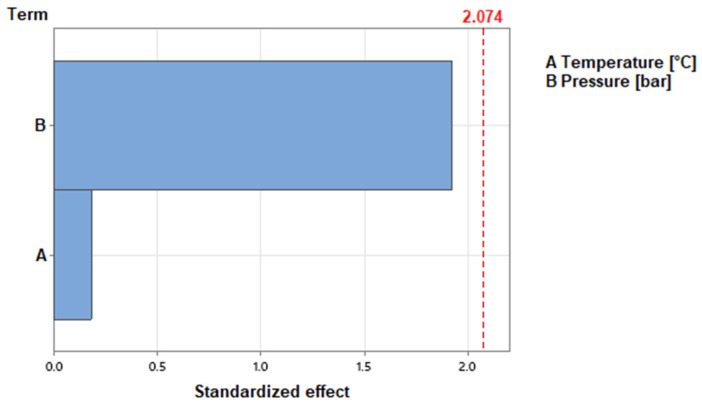
Standardized effects of the process parameters on void content, 95% confidence interval.

The results showed that the differences in void content are small for varying temperatures. For the investigated temperatures, the maximum variation is 3% between the EPs 250 °C and 280 °C. For varying pressures, the maximum variation occurs between the EPs 50 bar and 70 bar with a value of 5.7%. Again, small differences with overlaps in the standard deviations occur. Therefore, the parameter influences on the void content were examined for statistical relevance. The results using a confidence interval of 95% are provided in Figure 5. It is shown that for the produced CFRTP sheets, the void content is not influenced by the varied parameters in a statistically relevant manner.

### 3.2. Mechanical Properties

The mechanical properties of the PLA/flax composites were determined via tensile tests to investigate the influence of different temperatures and pressures. Therefore, the tensile modulus, the tensile strength and the elongation at break of ten samples per experimental point were examined. The results of the tensile tests are shown in Table 5.

**Figure 6:**
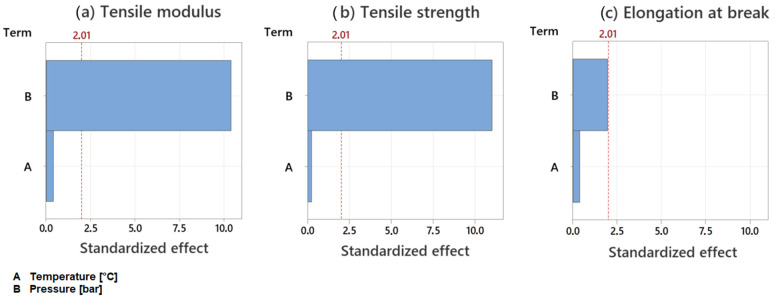
Standardized effects of the process parameters on mechanical properties, 95% confidence interval: (**a**) effect on tensile modulus; (**b**) effect on tensile strength; (**c**) effect on elongation at break.

The examination of the elongation at break did not show trends for varying temperatures or pressures. The results showed insignificant differences between the experimental points and partial overlaps in the standard deviations. Therefore, the influences of the process parameters on the mechanical properties were examined individually for statistical relevance. Figure 6 shows the standardized effects of temperature and pressure on the elongation at break, the tensile modulus and the tensile strength. The used confidence interval of 95% results in a threshold of statistical relevance of 2.01. It is visible that neither temperature nor pressure have a statistically relevant influence on the elongation at break.

The results for the remaining mechanical properties show mostly small differences for varying temperatures compared to the standard deviations. The only exception is the tensile modulus at 250 °C. The rising temperature from 220 °C to 250 °C does result in a decrease of 1414 N/mm^2^. The temperature change from 250 °C to 280 °C does result in an increase in the modulus of 1677 N/mm^2^. Figure 6 shows that none of the investigated properties are influenced by the temperature change in a statistically relevant manner.

Higher pressures result in a noticeable decrease in tensile modulus and tensile strength. For the change from 50 bar to 90 bar, the tensile modulus is reduced by 6800 N/mm^2^ and the tensile strength by 71 N/mm^2^ in total. This is confirmed in Figure 6, which shows that the influence of the pressure exceeds the threshold of statistical relevance. Given that the standardized effect is greater 10 while the threshold is set at 2.01, it is evident that the pressure influences the tensile modulus and the tensile strength significantly.

## 4. Discussion

The aim of this study was to determine the influence of impregnation temperature and process pressure on the mechanical properties of direct-extruded CFRTP sheets. The influence on fiber and void content was investigated as well because of the necessity of a high fiber and a low void content to achieve sufficient mechanical properties. According to the research, we derived that high temperatures help the impregnation process by reducing the melt viscosity. Therefore, the polymer can advance into the fabric more easily [14]. A high pressure is needed to achieve a full impregnation and consolidation, remove air and reduce the number of voids [13,15]. However, a significant influence of the process parameters on fiber and void content could not be identified for the investigated property ranges. The PLA exhibits a high viscosity. The applied pressure lowers the permeability of the fiber bundle significantly [13,17]. In combination, these two circumstances hinder micro-impregnation severely. This can be seen in microscopic images of the cut surface. The images exhibit areas where cavities arise because the fiber is only partially impregnated. It is assumed that entrapped air creates voids in the bundle. The shrinkage of the surrounding matrix material tears these voids open, leading to a larger cavity and therefore a higher void content in EP 2 [33,34]. A longer impregnation time can be considered for high-viscosity materials [13,14,17]. This means on the other hand that the fiber material experiences more thermal exposure, which can lower the mechanical properties significantly [29]. The optimal ratio between impregnation time and thermal exposure is specific for the combination of materials and remains yet to be found for PLA/flax fiber. Drying the fibers was not possible with this experimental setup but remains a promising approach to reduce the void content [35,36]. The manufacturer indicates 5–6% (by weight) of moisture inside the fabric. This was confirmed by TGA for the flax fibers. At high temperatures, this water evaporates and forms additional voids [23].

A parameter influence on the elongation at break could not be determined. The remaining mechanical properties were significantly lowered by applying a higher pressure. Because the fiber and void content were not influenced significantly by the changes in the parameters, it is suggested that the mechanical properties are not affected by the observed differences in fiber and void content. It is therefore assumed that the materials were influenced directly. The absence of a temperature influence suggests that the fibers were not thermally damaged. In comparison, the reduction in tensile modulus and tensile strength suggests damaged fibers due to high pressure. Especially, the change from 50 bar to 70 bar shows significantly different properties [13,37]. The elongation could not be influenced.

Most measurement techniques for the fiber and void content of bio-based composites are applicable for partwise production. Partwise production has the advantage that the fiber content is set and known beforehand [38,39,40]. To determine the unknown fiber content of a continuously produced composite, an alternative method was introduced. A major target of the method was to quantify changes in fiber and void content. The applied method comes with two challenges as it does not account for the fiber undulation and weft thread properly. This could be resolved by taking a second micrograph perpendicular to the first micrograph and combining the information. However, the measurement and evaluation efforts would significantly increase.

The applied design of experiments is designed to show the influences of single parameters. It is possible to extend the experiments to a full-factorial experimental plan to obtain further insight, e.g., into the interactions of process parameters and potentially the pressure, where fiber damage increases distinctly.

## 5. Conclusions

This study investigated the extrusion process for manufacturing CFRTP sheets from flax and PLA. It was shown that the properties of the produced PLA/flax fiber composites are influenced by the impregnation pressure. The results show that a higher pressure has a negative impact on the tensile modulus and the tensile strength. The best mechanical properties were achieved using an impregnation temperature of 250 °C and a pressure of 50 bar. To exploit the advantage of bio-based materials and their mechanical properties fully, future studies should be conducted to maximize fiber and minimize void content while applying a relatively low pressure, e.g., by using a matrix polymer with lower viscosity. Prolonging the impregnation time could be attempted, even though thermal exposure onto the fibers would increase. Drying prior to the processing could be an alternative option to decrease the moisture content and reduce the risk of voids originating from moisture residing inside the fabric. An improved process can be the basis for the highly economical manufacturing of bio-based and biodegradable CFRTP sheets. Through thermoforming, complex structural elements for interior automotive applications, e.g., door panels, dashboards, storage cabins, can be produced. These applications can reduce the total weight of the vehicle, therefore improving fuel economy and reducing CO_2_ emissions.

## Figures and Tables

**Table 1:** Thermal properties of PLA 2003D from the datasheet.

Thermal Properties	PLA 2003D	American Society for Testing and Materials (ASTM)
Melting point [°C]	145–160	D3418
Glass transition temperature [°C]	55.0–60.0	D3418
Deflection temperature at 0.46 MPa (66 psi) [°C]	55.0	E2092

**Table 2:** Experimental points for the direct extrusion.

Experimental Point	Temperature [°C]	Pressure [Bar]
1	250	70
2	250	50
3	250	90
4	220	70
5	280	70

**Table 3:** Comparison of the fiber and void content for the different evaluation methods, PP/glass fiber.

Method	Temperature [°C]	Pressure [bar]	Fiber Content [%]	Void Content [%]
Calcination	200	20	34.4	1.1
Calcination	260	40	46.5	5.7
Optical	200	20	35.3	2.2
Optical	260	40	46.7	5.9

**Table 4:** Results of fiber and void content analysis.

Temperature [°C]	Pressure [Bar]	Fiber Content [%]	Void Content [%]
220	70	33.8 ± 4.9	7.2 ± 1.3
250	70	31.1 ± 5.7	9.8 ± 1.9
280	70	34.8 ± 2.1	6.8 ± 0.6
250	50	28.8 ± 3.3	15.5 ± 1.6
250	90	32.6 ± 3.5	11.4 ± 0.7

**Table 5:** Results of the tensile tests, tensile modulus, tensile strength and elongation at break.

Temperature [°C]	Pressure [Bar]	Tensile Modulus [N/mm^2^]	Tensile Strength [N/mm^2^]	Elongation at Break [%]
220	70	8069 ± 469	69 ± 5	1.066 ± 0.098
250	70	6655 ± 431	66 ± 2	1.296 ± 0.123
280	70	8332 ± 432	67 ± 5	1.039 ± 0.133
250	50	13,407 ± 1003	129 ± 9	1.191 ± 0.123
250	90	6607 ± 650	58 ± 5	1.051 ± 0.149

## Data Availability

Data is contained within the article.
